# HGNChelper: identification and correction of invalid gene symbols for human and mouse

**DOI:** 10.12688/f1000research.28033.1

**Published:** 2020-12-21

**Authors:** Sehyun Oh, Jasmine Abdelnabi, Ragheed Al-Dulaimi, Ayush Aggarwal, Marcel Ramos, Sean Davis, Markus Riester, Levi Waldron

**Affiliations:** 1 Epidemiology and Biostatistics, Graduate School of Public Health and Health Policy, City University of New York, New York, 10027, USA; 2 Institute for Implementation Science and Population Health, New York, 10027, USA; 3 School of Medicine, University of Utah, Utah, 84132, USA; 4 CSIR-Institute of Genomics and Integrative Biology, New Delhi, 110025, India; 5 Academy of Scientific and Innovative Research, Ghaziabad, Uttar Pradesh, 201 002, India; 6 Center for Cancer Research, National Cancer Institute, Maryland, 20892, USA; 7 Novartis Institutes for BioMedical Research Incorporation, Massachusetts, 02139, USA

**Keywords:** gene symbols, molecular biology, HGNC, MGI

## Abstract

Gene symbols are recognizable identifiers for gene names but are unstable and error-prone due to aliasing, manual entry, and unintentional conversion by spreadsheets to date format. Official gene symbol resources such as HUGO Gene Nomenclature Committee (HGNC) for human genes and the Mouse Genome Informatics project (MGI) for mouse genes provide authoritative sources of valid, aliased, and outdated symbols, but lack a programmatic interface and correction of symbols converted by spreadsheets. We present HGNChelper, an R package that identifies known aliases and outdated gene symbols based on the HGNC human and MGI mouse gene symbol databases, in addition to common mislabeling introduced by spreadsheets, and provides corrections where possible. HGNChelper identified invalid gene symbols in the most recent Molecular Signatures Database (mSigDB 7.0) and in platform annotation files of the Gene Expression Omnibus, with prevalence ranging from ~3% in recent platforms to 30-40% in the earliest platforms from 2002-03. HGNChelper is installable from CRAN.

## Introduction

Gene symbols are widely used in biomedical research because they provide descriptive and memorable nomenclature for communication. However, gene symbols are constantly updated through the discoveries and re-identification of genes, resulting in new names or aliases. For example, *GCN5L2* ( *G*eneral *C*ontrol of amino acid synthesis protein *5-L*ike *2*) is a gene symbol that was later discovered to function as a histone acetyltransferase and therefore renamed as *KAT2A* ( *K*(lysine) *A*cetyl *T*ransferase *2A*)) ^1^. In addition to the rapid and constant updates on valid gene symbols, commonly used spreadsheet software, such as Microsoft Excel, modify some gene symbols, converting them into dates or floating-points numbers ^2, 3^. For example, ‘ *DEC1*’, a symbol for ‘ *D*eletion in *E*sophageal *C*ancer *1*’ gene, can be exported in date format, ‘1-DEC’. There have been attempts to rectify gene symbol issues, but they have largely been limited to Excel-modified gene symbols. Also the suggested solutions often reference static files with the corrections curated at the time of publication ^3^ or comprise scripts for detecting the existence of Excel-modified gene symbols without correction ^2^. In recognition of the importance of the spreadsheet modification issues, HGNC recently announced that all symbols that auto-convert to dates in Excel have been changed ^4^. However, much literature and public data still contains outdated and incorrect gene symbols, motivating a convenient method of systematic detection and correction. To systematically identify historical aliases, correct for capitalization differences, and simultaneously correct spreadsheet-modified gene symbols, we built the HGNChelper R package. HGNChelper maps different aliases and spreadsheet-modified gene symbols to approved gene symbols maintained by The HUGO Gene Nomenclature Committee (HGNC) database ^5^. HGNChelper also supports mouse gene symbol correction based on the Mouse Genome Informatics (MGI) database ^6^.

## Methods

### Implementation

***Source data.*** Human gene symbols are accessed from HGNC Database ftp site ( ftp://ftp.ebi.ac.uk/pub/databases/genenames/new/tsv/hgnc_complete_set.txt) ^7^ and mouse gene symbols are acquired from MGI Database ( http://www.informatics.jax.org/downloads/reports/MGI_EntrezGene.rpt) ^6^. These URLs, and their access and processing, are handled by HGNChelper so the user does not interact directly with them.

***Algorithm.*** Human gene symbol correction is processed in three steps. First, capitalization is fixed: all letters are converted to upper-case, except the open reading frame (orf) nomenclature, which is written in lower-case. Second, dates or floating-point numbers generated via Excel-modification are corrected using a custom index generated by importing all human gene symbols into Excel, exporting them in all available date formats, and collecting any gene symbols that are different from the originals. In the last and most commonly applied step, aliases are updated to approved gene symbols in the HGNC database. Mouse gene symbol correction follows the same three steps as in human gene symbol correction, except the capitalization step since mouse gene symbols begin with an uppercase character, followed by all lowercase.

***User interface.*** The user interface of HGNChelper does not include any local input or output files; instead it uses R data structures as function arguments and output. Base R data export functions such as write.table can be used to write results to file in whichever format required. The input arguments to the main function, checkGeneSymbols, are:

1. **x**: A character vector of gene symbols to check for modified or outdated values
2. **chromosome**: An optional integer vector the same length as x, providing chromosome numbers for each gene
3. **unmapped.as.na**: A logical value, if TRUE (default), unmapped symbols will appear as NA in the Suggested.Symbol output column. If FALSE, the original unmapped symbol will be kept.
4. **map**: An optional user-updated or non-standard gene map. The default maps can be updated by running the interactive example provided in the help page to checkGeneSymbols.
5. **species:** A required character vector of length 1, either "human" (default) or "mouse".

checkGeneSymbols returns an R data.frame with one row per input gene and three columns:

1. The first column of the data frame shows the input gene symbols.
2. The second column indicates whether the input symbols are valid
3. The third column provides a corrected gene symbol where possible.
A message is printed indicating when the package’s built-in map was last updated. Because the gene symbol databases are updated as frequently as every day, we provide the getCurrentHumanMap and getCurrentMouseMap functions for updating the reference map without requiring an HGNChelper software update. These functions fetch the most up-to-date version of the map from HGNC and MGI, respectively, and users can provide the output of these functions through the map argument of checkGeneSymbols function. However, fetching a new map requires internet access and takes longer than using the package’s built-in index.

### Operation

HGNChelper is an R package installable from CRAN on Linux, Windows, and OSX. It requires a base installation of R (> 3.5.0) and no other dependencies, and has minimal hardware requirements that should be met by any computer capable of installing the R dependency.

## Results

**Figure 1.**
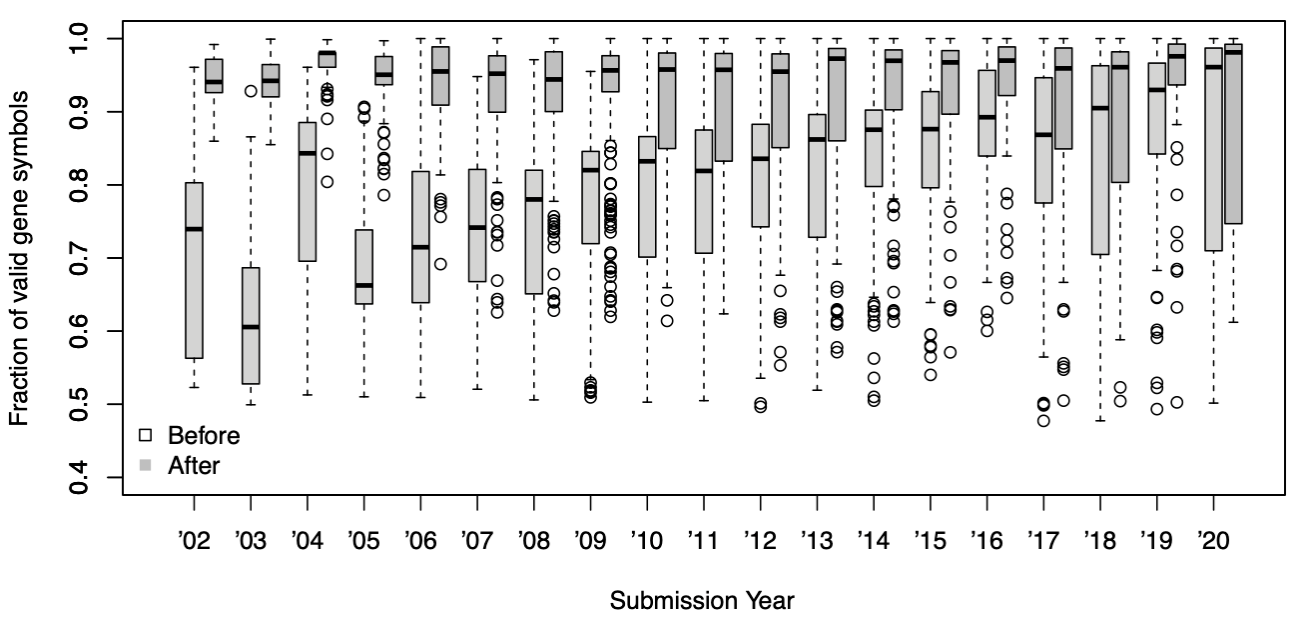
The fraction of valid gene symbols in GPL files grouped by year of data submission. Each dot represents a unique GPL. Older entries show a smaller fraction of valid gene symbols than more recent entries (Before, white box), but many of which are successfully corrected by HGNChelper (After, grey box).

To evaluate the performance of HGNChelper, we quantified the extent of invalid gene symbols present in platform annotation files in the Gene Expression Omnibus (GEO) database from 2002 to 2020. We downloaded 20,716 GEO platform annotation (GPL) files using GEOquery::getGEO ^8^, of which 2,044 platforms were suspected to contain gene symbol information based on matching to valid symbols. There is a clear trend of increasing proportion of invalid gene symbols with age of platform submission ( Figure 1), ranging from an average of ~3% for recent platforms and increasing with age to ~20% in 2010 and 30–40% in the earliest platforms from 2002–03. The overall proportion of valid gene symbols was 79%, increasing to 92% after HGNChelper correction. The 8% remaining, invalid gene symbols were mostly long non-coding RNA (lncRNA), pseudogenes, commercial product IDs such as probe ID, missing data, and gene symbols from non-human species, erroneously included together with human gene symbols. We also checked the validity of gene symbols in the Molecular Signatures Database (MSigDB 7.0) ^9^. Out of 38,040 gene symbols used in MSigDB version 7.0, 850 were invalid, and this number reduces to 453 after HGNChelper correction, of which the majority were lncRNA and a few withdrawn symbols.

## Discussion

Gene symbols are error-prone and unstable, but remain in common use for their memorability and interpretability. Our analysis of public databases containing gene symbols emphasizes the need for gene symbol correction particularly when using symbols from older datasets and reported results. Such correction should be routinely done when gene symbols are part of high-throughput analysis, such as re-analysis of targeted gene panels for precision medicine, which tend to be annotated with gene symbols (e.g. 10), in Gene Set Enrichment Analysis using the gene symbol versions of popular databases such as MSigDB ^9^ or GeneSigDB ^11^, or when performing systematic review or meta-analysis of published multi-gene signatures (e.g. 12). HGNChelper implements a programmatic and straightforward approach to the routine identification and correction of invalid gene symbols.

## Software availability

Package available from CRAN: https://cran.r-project.org/package=HGNChelper

Source code available from: https://github.com/waldronlab/HGNChelper/

Archived source code as at time of publication: https://doi.org/10.5281/zenodo.4309985 ^13^

License: GPL (≥ 2.0)
