# Supplementary material for: HGNChelper: identification and correction of invalid gene symbols for human and mouse
Source: F1000Res. 2022 Jun 9;9:1493. Originally published 2020 Dec 21. [Version 2] doi: 10.12688/f1000research.28033.2 (PMC7856679; doi:10.12688/f1000research.28033.2)
Supplement: Peer review history

# Peer review: Reviewer response for version 2

**DOI:** 10.5256/f1000research.133588.r140199

**Published:** 2022-06-27

**Authors:** Susan Tweedie

**Affiliations:** 1 HUGO Gene Nomenclature Committee (HGNC), European Molecular Biology Laboratory, European Bioinformatics Institute, Hinxton, UK

The authors have addressed my major concern by adding a Limitations section.

Are the conclusions about the tool and its performance adequately supported by the findings presented in the article?

Partly

Is the rationale for developing the new software tool clearly explained?

Partly

Is the description of the software tool technically sound?

Partly

Are sufficient details of the code, methods and analysis (if applicable) provided to allow replication of the software development and its use by others?

Yes

Is sufficient information provided to allow interpretation of the expected output datasets and any results generated using the tool?

Partly

Reviewer Expertise:

Gene nomenclature, Genomics

I confirm that I have read this submission and believe that I have an appropriate level of expertise to confirm that it is of an acceptable scientific standard.

# Peer review: Reviewer response for version 1

**DOI:** 10.5256/f1000research.31006.r76417

**Published:** 2021-02-02

**Authors:** Marcin Cieślik

**Affiliations:** 1 Michigan Center for Translational Pathology, University of Michigan Medical School, Ann Arbor, USA

HGNChelper is a particularly valuable tool in the toolbox of a bioinformatics practicioneer. It addresses a real problem, which while superficially trivial, actually affects the quality of analyses. 

 I use HGNChelper ALL THE TIME especially if a dataset ends up being garbled by Excel. It is easy to use, in most recent versions reasonably fast, and frankly just gets the job done

 The paper is a short description of the motivation and implementation. With a short study detailing the evolution of gene symbolss.

 What I miss in the paper is an assessment of HGNChelper failures (to increase my confidence in the tool). For example how often are symbols converted incorrectly because the same gene symbol was used, at different times, to denote different genes.

Are the conclusions about the tool and its performance adequately supported by the findings presented in the article?

Partly

Is the rationale for developing the new software tool clearly explained?

Yes

Is the description of the software tool technically sound?

Yes

Are sufficient details of the code, methods and analysis (if applicable) provided to allow replication of the software development and its use by others?

Yes

Is sufficient information provided to allow interpretation of the expected output datasets and any results generated using the tool?

Yes

Reviewer Expertise:

Cancer Genetics

I confirm that I have read this submission and believe that I have an appropriate level of expertise to confirm that it is of an acceptable scientific standard.

# Response

**Published:** 2022-04-27

**Authors:** Levi Waldron

**Affiliations:** CUNY Graduate School of Public Health and Health Policy, USA

Thank you for reviewing our manuscript and for your encouraging comments. 

**Comment 1: The main issue raised is when HGNChelper fails to map symbols, it is important for users to understand the limitations of what the tool can and cannot map.**

 To address this point we manually reviewed many cases where HGNChelper correction efficiency is low in Figure 1 and almost all unmapped symbols fell into one of the following categories:

1. Long non-coding RNAs (e.g. “lnc-ARMCX4-1”, “lnc-SOX11-1”)
2. Withdrawn symbol (e.g. “OCLM”)
3. Uncharacterized gene (e.g. “LOC644669”): Symbols beginning with LOC. When a published symbol is not available, and orthologs have not yet been determined, this may be represented as ‘LOC’ + the GeneID. 
4. Non-human gene symbol
5. Missing data
6. Commercial  product name (e.g. Probe ID)

We have added this to the manuscript section “Limitations”.

# Peer review: Reviewer response for version 1

**DOI:** 10.5256/f1000research.31006.r76418

**Published:** 2021-01-20

**Authors:** Susan Tweedie

**Affiliations:** 1 HUGO Gene Nomenclature Committee (HGNC), European Molecular Biology Laboratory, European Bioinformatics Institute, Hinxton, UK

The paper describes an R package for that checks whether human and mouse symbols match an HGNC or MGI approved symbol and if not suggests a replacement by, correction of capitalization, correction of Excel date and floating-point transformations and matching to alias symbols.

 The rationale for developing the new software tool is generally clearly explained. As the authors point out, symbols do change (although the HGNC are now committed to making as few symbol changes as possible) and there is a need to check symbols are valid. As the human symbols that convert to dates in Excel have all been changed, this should be less of a problem going forward. However, these mangled symbols persist in historic data sets and some authors will undoubtedly continue to use problematic aliases such as OCT3 so that aspect of the tool is helpful. It may be worth adding that conversion of gene symbols to floating-point numbers in Excel is more of an issue for mouse genes with RIKEN identifiers than human gene symbols.

 The authors should also address whether there are any other R packages that have similar functionality. The name HGNChelper could lead some to think this is an HGNC endorsed tool; given that a symbol checking tool is already available from the HGNC ( https://www.genenames.org/tools/multi-symbol-checker/) (albeit not an R package) the authors should mention this exists and ideally compare the functionality of their tool versus the HGNC tool.

 While this is likely to be a useful tool for R users it should come with a few words of caution given that you cannot always be completely sure which gene a symbol refers to in the absence of confirmation via an ID or other additional information. For example, FHL1 is both an approved symbol and an alias of CFH so while FHL1 is a valid symbol the input data may refer to CFH. There are also cases where a symbol is an alias for several genes but not an approved symbol itself e.g. NIP (or Nip) which not an approved symbol but is an alias of GIPC1, DUOXA1, and CRPPA. The authors should clarify how the algorithm deals with cases where an input symbol matches more than one gene. This would address my concerns about whether this is sufficient information provided to allow interpretation of the expected output datasets and any results generated using the tool.

 I note that the input contains optional chromosome information but there is no mention of how this is used – does the algorithm take this information into account when determining whether a symbol is valid or not?

 The authors note that some symbols in their test that could not be updated were lncRNAs and pseudogenes. As both of these classes of gene are named by HGNC it would be good to expand on why the tool failed with these genes – do these particular genes lack an HGNC symbol?

 Figure 1 is hard to interpret - it is unclear what the white boxes mentioned in the legend refer to. Improving the clarity of the figure would address my concerns about whether the conclusions about the tool and its performance adequately supported by the findings presented in the article.

Are the conclusions about the tool and its performance adequately supported by the findings presented in the article?

Partly

Is the rationale for developing the new software tool clearly explained?

Partly

Is the description of the software tool technically sound?

Partly

Are sufficient details of the code, methods and analysis (if applicable) provided to allow replication of the software development and its use by others?

Yes

Is sufficient information provided to allow interpretation of the expected output datasets and any results generated using the tool?

Partly

Reviewer Expertise:

Gene nomenclature, Genomics

I confirm that I have read this submission and believe that I have an appropriate level of expertise to confirm that it is of an acceptable scientific standard, however I have significant reservations, as outlined above.

# Response

**Published:** 2022-04-27

**Authors:** Levi Waldron

**Affiliations:** CUNY Graduate School of Public Health and Health Policy, USA

Thank you for reviewing our manuscript and for your constructive comments. Below are our responses to the individual comments.

**Comment 1: It may be worth adding that conversion of gene symbols to floating-point numbers in Excel is more of an issue for mouse genes with RIKEN identifiers than human gene symbols.**

 The reviewer is correct that human gene symbols prone to Excel conversion have now been changed, but many still exist in the literature and public databases as demonstrated in Figure 1.  HGNChelper does not currently fix RIKEN identifiers, so we don’t draw this comparison in the manuscript.

**Comment 2: The authors should also address whether there are any other R packages that have similar functionality. The name HGNChelper could lead some to think this is an HGNC endorsed tool; given that a symbol checking tool is already available from the HGNC (https://www.genenames.org/tools/multi-symbol-checker/) (albeit not an R package) the authors should mention this exists and ideally compare the functionality of their tool versus the HGNC tool.**

 We now compare HGNChelper with the function alias2Symbol from the limma package. This is described in the response to comment 1 from Reviewer 1. 

 Thank you for pointing us to the HGNC’s own tool, the Multi-symbol checker. We understand that our package name, HGNChelper, can potentially imply the endorsement from HGNC, so we clarified in the manuscript that Multi-symbol checker is the tool supported by HGNC. We also compared the HGNChelper and Multi-symbol checker from HGNC. Here are the major points that differentiate these tools:

1. Implementation: Multi-symbol checker is a web-based UI tool. Users can provide an input as a comma- or space- separated list of gene symbols, directly typing-in or uploading the file. Outputs are displayed in the interface as a sortable table and users can choose to download it as a csv file. HGNChelper is a R package, which takes an input as a character vector and outputs the result as a data frame, which can be saved and exported in a different format, such as csv, tsv, rds, etc. 
2. Excel-modified gene symbols: HGNChelper corrects inputs in a potentially excel-modified format (e.g. “9/7”, “1-Mar”, “Oct4”), and suggest the original symbol (e.g. “SEPTIN7”, “MARCHF1 /// MTARC1”, “POU5F1”). This functionality is not part of multi-symbol checker - it marks them as ‘Unmatched’ as with any other unmatchable symbol. 
3. Chromosome location: Multi-symbol checker provides the chromosome location as a part of the default output, if the approved symbol is available for a given input. HGNChelper provides the chromosome information only if it is provided with the input gene symbol - it validates whether the input chromosome information is correct or not, and if it’s wrong, gives the correct chromosome location. 

**Comment 3: While this is likely to be a useful tool for R users it should come with a few words of caution given that you cannot always be completely sure which gene a symbol refers to in the absence of confirmation via an ID or other additional information. For example, FHL1 is both an approved symbol and an alias of CFH so while FHL1 is a valid symbol the input data may refer to CFH.**

 In this example, FHL1 will be returned as a valid symbol, unless the chromosome of CFH is specified. For example:

 > checkGeneSymbols("FHL1")

      x Approved Suggested.Symbol

 1 FHL1     TRUE             FHL1

 > checkGeneSymbols(c("FHL1", "FHL1"), chromosome = c("X", "1"))

      x Approved Suggested.Symbol Input.chromosome Correct.chromosome

 1 FHL1     TRUE             FHL1                X                  X

 2 FHL1    FALSE              CFH                1                  1

 We have added this discussion to the “Limitations” section. 

**Comment 4: There are also cases where a symbol is an alias for several genes but not an approved symbol itself e.g. NIP (or Nip) which is not an approved symbol but is an alias of GIPC1, DUOXA1, and CRPPA. The authors should clarify how the algorithm deals with cases where an input symbol matches more than one gene. This would address my concerns about whether this is sufficient information provided to allow interpretation of the expected output datasets and any results generated using the tool.**

 When an input symbol matches ambiguously to more than one gene, HGNChelper displays all the matching genes. For example, HGNChelper::checkGeneSymbols(“NIP”) will return “CRPPA /// DUOXA1 /// GIPC1'' as the suggested symbols.

- If there is only one valid gene symbol matched with the input, HGNChelper simply evaluates whether the provided chromosome information is correct or not, and if it’s incorrect, outputs the correct chromosome location under the ‘Correct.chromosome’ column. For example, HGNChelper::checkGeneSymbols(“NIP”, chromosome = 1) will return “CRPPA /// DUOXA1 /// GIPC1'' as the suggested symbol and “7 /// 15 /// 19'' as the correct chromosome.
- If the input matches more than one gene, the chromosome information is used to specify the suggested gene symbol. For example,  HGNChelper::checkGeneSymbols(“NIP”, chromosome = 7) will return “CRPPA'' as the suggested symbol and “7'' as the correct chromosome.
- lncRNAs and pseudogenes can be updated as long as they are not ‘uncharacterized genes’, whose symbols start with ‘LOC’. Based on NCBI, when a published symbol is not available and orthologs have not yet been determined, gene will provide a symbol that is constructed as ‘LOC’ + the GeneID. So HGNChelper can not update them because there are no approved gene symbols for them. 

**Comment 5: Figure 1 - Improve the clarity of the figure**

 We apologize for the confusing color display. Color schema for Figure 1 is fixed in the updated manuscript.

# Peer review: Reviewer response for version 1

**DOI:** 10.5256/f1000research.31006.r76531

**Published:** 2021-01-18

**Authors:** Mikhail G. Dozmorov

**Affiliations:** 1 Biostatistics Department, Virginia Commonwealth University, Richmond, VA, USA

The manuscript "HGNChelper: identification and correction of invalid gene symbols for human and mouse" by Oh S. et al. describes the HGNChelper R package that corrects the common problem of misformatted gene symbols and aliases. The package works with both human and mouse gene symbols. The manuscript describes the procedure for obtaining current gene symbols and creating a mapping between the correct and invalid gene symbols. Analysis of >20K GEO datasets demonstrated the pervasive presence of invalid gene symbols, with the proportion of such symbols decreasing over time. Applied to the recent version of MSigDB, it shows the presence of 850 invalid gene symbols; half of them can be corrected with HGNChelper. The manuscript is well-written, and the package is made using R best practices. 

 Minor comments about the manuscript include:

- The limma R package has the alias2Symbol function with similar functionality. How the functionality of HGNChelper differs or improves upon this function?
- Figure 1 - Before/after boxplots look identical in the black and white version. Please, correct.

The following comments about the package interface are suggestive.

- The current output of the checkGeneSymbols() function returns a data frame with three columns (x, Approved, Suggested.Symbol). Suggesting including an argument "simplify" (TRUE by default) that will return one vector of the same length and order as the original vector of gene symbols, with NAs replacing non-mappable symbols. The rationale is to use this function as a wrapper around the original vector of gene symbols, e.g., checkGeneSymbols(my_genes), returning a drop-in replacement vector of corrected gene symbols. An example is the p.adjust() function that, given a vector of p-values, returns a vector of p-values corrected for multiple testing.
- The package provides separate functions to update gene maps. These updated gene maps can then be used in the checkGeneSymbols() function. Suggesting including an argument "update.map" (FALSE by default) to checkGeneSymbols(), that, if TRUE, will automatically update gene maps.

Are the conclusions about the tool and its performance adequately supported by the findings presented in the article?

Yes

Is the rationale for developing the new software tool clearly explained?

Yes

Is the description of the software tool technically sound?

Yes

Are sufficient details of the code, methods and analysis (if applicable) provided to allow replication of the software development and its use by others?

Yes

Is sufficient information provided to allow interpretation of the expected output datasets and any results generated using the tool?

Yes

Reviewer Expertise:

Bioinformatics, genomics

I confirm that I have read this submission and believe that I have an appropriate level of expertise to confirm that it is of an acceptable scientific standard.

# Response

**Published:** 2022-04-27

**Authors:** Levi Waldron

**Affiliations:** CUNY Graduate School of Public Health and Health Policy, USA

Thank you for reviewing our manuscript and for your constructive comments. Below are our responses to the individual comments.

**Comment 1: The limma R package has the alias2Symbol function with similar functionality. How does the functionality of HGNChelper differ or improve upon this function?**

 The biggest difference is that limma::alias2Symbol and limma::alias2SymbolTable are intended only to translate known gene aliases, whereas HGNChelper is intended for heterogeneous input that may include aliases, valid symbols, Excel-modified symbols, incorrect capitalization, and unmappable symbols, and to provide a map between input and output. limma::alias2SymbolTable maintains the length of the output vector as same as the input, but if there are multiple aliases, it displays only the one with the lowest Entrez ID number, whereas HGNChelper returns a delimited vector of all aliases. The following example demonstrates these differences:

 > library(HGNChelper)

 > input = c("FN1", "TP53", "UNKNOWNGENE",

 +          "7-Sep", "9/7", "1-Mar", 

 +          "Oct4", "4-Oct", "OCT4-PG4", 

 +          "C19ORF71", "C19orf71", "NIP")

 > checkGeneSymbols(input)

 Maps last updated on: Mon Sep 28 18:31:21 2020

              x Approved           Suggested.Symbol

 1          FN1     TRUE                        FN1

 2         TP53     TRUE                       TP53

 3  UNKNOWNGENE    FALSE                       

 4        7-Sep    FALSE                    SEPTIN7

 5          9/7    FALSE                    SEPTIN7

 6        1-Mar    FALSE         MARCHF1 /// MTARC1

 7         Oct4    FALSE                     POU5F1

 8        4-Oct    FALSE                     POU5F1

 9     OCT4-PG4    FALSE                   POU5F1P4

 10    C19ORF71    FALSE                   C19orf71

 11    C19orf71     TRUE                   C19orf71

 12         NIP    FALSE CRPPA /// DUOXA1 /// GIPC1

 Warning messages:

 1: In checkGeneSymbols(input) :

  Human gene symbols should be all upper-case except for the 'orf' in open reading frames. The case of some letters was corrected.

 2: In checkGeneSymbols(input) : x contains non-approved gene symbols

 > library(limma)

 > library(org.Hs.eg.db)

 > alias2Symbol(alias = input)

 [1] "FN1"      "TP53"     "C19orf71" "GIPC1"    "DUOXA1"  

 > alias2SymbolTable(alias = input)

  [1] "FN1"      "TP53"     NA         NA         NA         NA        

  [7] NA         NA         NA         NA         "C19orf71" "GIPC1"   

 Warning message:

 In alias2SymbolTable(alias = input) :

  Multiple symbols ignored for one or more aliases

 Additionally, limma::alias2Symbol uses Bioconductor org*.db packages to map aliases for multiple organisms. org*.db packages in turn pull data from NCBI and update it with each Bioconductor release. HGNChelper is a CRAN package with no dependency on non-base packages, and instead downloads aliases directly from the HUGO and MGI projects. limma::alias2Symbol however provides an advantage of supporting any organism for which an org*.db package is available, whereas HGNChelper supports only human and mouse.

**Comment 2: Figure 1 - Before/after boxplots look identical in the black and white version. Please, correct.**

 We apologize for the confusing color display. The color schema for Figure 1 is fixed in the updated manuscript. 

**Comment 3: The current output of the checkGeneSymbols() function returns a data frame with three columns (x, Approved, Suggested.Symbol). Suggesting including an argument "simplify" (TRUE by default) that will return one vector of the same length and order as the original vector of gene symbols, with NAs replacing non-mappable symbols. The rationale is to use this function as a wrapper around the original vector of gene symbols, e.g., checkGeneSymbols(my_genes), returning a drop-in replacement vector of corrected gene symbols. An example is the p.adjust() function that, given a vector of p-values, returns a vector of p-values corrected for multiple testing.**

 This is a good use case, but we are reluctant to allow a function argument to change the class (and the contract) of what the function returns. Motivated by arguments for “Type consistency” such as by Gillespie and Lovelace (Efficient R programming, https://csgillespie.github.io/efficientR/, section 3.5.2), we think it is less error-prone to require a simple but explicit step to change data class. We’ve added an example to the checkGeneSymbols help page to provide a straightforward solution in this use case:

 > human

  [1] "FN1"         "TP53"        "UNKNOWNGENE" "7-Sep"       "9/7"         "1-Mar"      

  [7] "Oct4"        "4-Oct"       "OCT4-PG4"    "C19ORF71"    "C19orf71"   

 > checkGeneSymbols(human)$Suggested.Symbol

 Maps last updated on: Thu Mar 25 08:36:49 2021

  [1] "FN1"                "TP53"               NA                   "SEPTIN7"           

  [5] "SEPTIN7"            "MARCHF1 /// MTARC1" "POU5F1"             "POU5F1"            

  [9] "POU5F1P4"           "C19orf71"           "C19orf71"          

 Warning messages:

 1: In checkGeneSymbols(human) :

   Human gene symbols should be all upper-case except for the 'orf' in open reading frames. The case of some letters was corrected.

 2: In checkGeneSymbols(human) : x contains non-approved gene symbols

**Comment 4: The package provides separate functions to update gene maps. These updated gene maps can then be used in the checkGeneSymbols() function. Suggesting including an argument "update.map" (FALSE by default) to checkGeneSymbols(), that, if TRUE, will automatically update gene maps.**

 We are reluctant to add an argument that automatically updates the data resource first because results from the same code and the same version of HGNChelper would produce different results at any time since the HGNC and MGI databases change frequently, and secondly because the potential for unnecessary heavy load on the HGNC database could result in restrictions on the bulk downloads we rely on. To maintain reproducibility by default we require the user to download and save the map if they want a version newer than what HGNChelper has, an approach also compatible with caching programs like BiocFileCache. We have added the following explanation to the vignette under the title, “Updating maps of aliased gene symbols”:

*We intentionally avoid automatic update of the map to maintain reproducibility, because the same code from the same version of HGNChelper could produce different results at any time with automatic map update.*
